# Daily activity patterns in older adults receiving initial support: the association between daily steps and sitting in bouts of at least 60 min

**DOI:** 10.1186/s12877-024-04681-3

**Published:** 2024-01-23

**Authors:** Joakim Niklasson, Cecilia Fagerström, Sofia Backåberg, Terese Lindberg, Patrick Bergman

**Affiliations:** 1 Faculty of Health and Life Sciences, Linnaeus University, Kalmar, Sweden; 2 Department of Research, Region Kalmar County, Kalmar, Sweden; 3 Faculty of Kinesiology, University of Calgary, Calgary, Canada; 4 Department of Health, Blekinge Institute of Technology, Karlskrona, Sweden; 5 Faculty of Health and Life Sciences, Department of Medicine and Optometry, Linnaeus University, eHealth Institute, Kalmar, Sweden

**Keywords:** Accelerometer, Aging, Physical activity, Sedentary behavior, Sitting, Walking independence

## Abstract

**Background:**

Aging has a significant impact on health, underlining the importance of maintaining physical function and reducing time spent sitting among older adults. To understand how to reduce prolonged sitting or increase physical activity, factors related to the daily living and observed daily activity patterns should be explored. This study aimed to investigate the association between daily steps, self-rated health, physical activity, sedentary behavior, motivation to exercise and fear of falling among older adults receiving initial support.

**Method:**

Cross-sectional design with total population questionnaire data from adults aged ≥ 60 years (*n* = 917), living at home with initial support from municipal care in southern Sweden. The older adults were offered to participate in a follow-up study measuring daily activity patterns with accelerometers (*n* = 72). Linear regression was used to analyze associations between daily steps and possible predictors.

**Results:**

The linear model (${R}^{2}=$0.478) showed that sitting in unbroken bouts of > 60 min (β = -0.313, *p* < 0.05), walking independently outdoors (β = 0.301, *p* < 0.05), intending to increase physical activity (β = -0.294, *p* < 0.05), sex (β = 0.279, *p* < 0.05), relative autonomy index (β = 0.258, *p* < 0.05), fear of falling (β = -0.238, *p* < 0.05), and self-rated health (β = 0.213, *p* < 0.05) predicted daily steps.

**Conclusion:**

The model of predictors brings new understanding regarding daily steps among community-dwelling older adults. The association between sitting in bouts of > 60 min and daily steps is interesting as 35% of participants had a number of sitting bouts that on average, showed 30% less steps taken. Minimizing long sitting bouts and maintaining physical functioning to promote independence when walking outdoors can be tools for clinical practitioners devising interventions to break prolonged sitting among community-dwelling older adults. Future research should prioritize studying older adults’ outdoor walking independence, including its relation to walking with or without assistive devices and its impact on physical activity and sedentary behavior.

**Supplementary Information:**

The online version contains supplementary material available at 10.1186/s12877-024-04681-3.

## Background

Through continuous development of health care, the possibility to prevent, treat, and cure diseases has increased. As a result, the number and proportion of adults over 60 years is increasing dramatically, leading to a demographic shift with more older adults and fewer people able to care for them. Thus, strategies to promote healthy aging are needed [1]. Becoming older has an impact on functional capability and preserving physical functionality is important for healthy aging [1, 2]. Aging affects functional, biological, social, and psychological aspects, which makes the process non-linear and complex [3]. With the connectivity to these aspects ageing is expected to impact daily steps and the capability of engaging in physical activity of higher intensity [4].. The complexity of aging and lacking motivation for physical activity in daily life are two of the most frequently cited barriers to older adults’ engagement in physical exercise [5–7]. When addressing physical activity behaviors, there is a need to understand the impact that self-determination and motivation regulation for physical activity have on older adults’ daily activity patterns [8]. Being motivated to be physically active is governed by both internal and external rewards. Understanding the internal motivation known as the inner drive, or intrinsic regulation, is of great importance when addressing physical activity behaviors among older adults [9].

Sedentary behavior and physical inactivity are important modifiable risk factors related to a range of health conditions, including mortality [10–14]. The daily activity patterns of older adults vary, but time spent sitting accumulates to more than 10 h per day on average [15, 16]. Sedentary behavior is characterized by low energy expenditure and is defined as any waking behavior while sitting or lying down [17]. Furthermore, for community-dwelling older adults living in a municipality in southern Sweden, being sedentary also means having a lack of physical activity and social interactions [18]. Reducing time spent sitting and performing at least 150 min of moderate to vigorous intensity physical activity per week are two of the recommendations given for healthy aging, but the challenge to increasing physical activity remains [2]. In a literature review by Katzmarzyk, Powell [19], evidence showed that previous studies mainly observed and measured study participants’ physical activity levels, as it is hard to properly quantify time spent sedentary. However, focusing on quantification of risk related to being sedentary was recommended and the question of how to change long-term behavior remains unanswered [19].

Fear of falling has a great impact on older adults’ health and ability to walk independently. According to Liu, Hou [20], having had multiple falls has been shown to be a predictor of interest regarding the ability to independently carry out daily activities, making it an important factor in understanding older adults’ daily activity patterns. Recent research indicates that there is a need to address a broader spectrum of activity when supporting older adults to become more physically active and less sedentary [21, 22]. According to Walker, Greenwood-Hickman [23], this broadening of the activity spectrum results in the inclusion of physical activity below moderate intensity when referring to increasing functional capability, and underlines the importance of not overlooking daily steps when observing activity patterns among older adults aged 75 years or older. Daily steps have been related to positive health outcomes, but increased evidence for the inverse relationship have been found recently [24]. This makes health an important measure to include when studying sedentary behavior.

Returning to the findings of Walker, Greenwood-Hickman [23], there is still much to learn from investigating step count in older adults, such as its role in future interventions aimed to change sedentary behavior towards more physically active ones. By increasing daily steps, time spent sitting can be reduced. According to Paluch, Bajpai [25], step count can be used as a recommendation in interventions aiming to promote health. However, before making interventions, there is a need to understand underlying behaviors and predictors of daily steps.

## Method

### Aim

This study aimed to investigate the association between daily steps and self-rated health, physical activity, sedentary behavior, motivation for exercise, and fear of falling among older adults receiving initial support.

### Study design

Cross-sectional study of questionnaire and accelerometer data.

### Participants and recruitment procedure

The study population consisted of community-dwelling residents of a municipality in Sweden, aged 60 years and above, who were participants in the baseline (2018–2019) of the total population questionnaire *Sedentary behavior in older adults and supportive methods to promote healthy aging*. Out of 1,617 eligible residents, 917 answered the questionnaire. The questionnaire was conducted in a region encompassing a diversity of small towns and rural areas, where 24% of citizens were 65 years or older and 11.5% were receiving support from municipal caregivers [26].

**Fig. 1:**
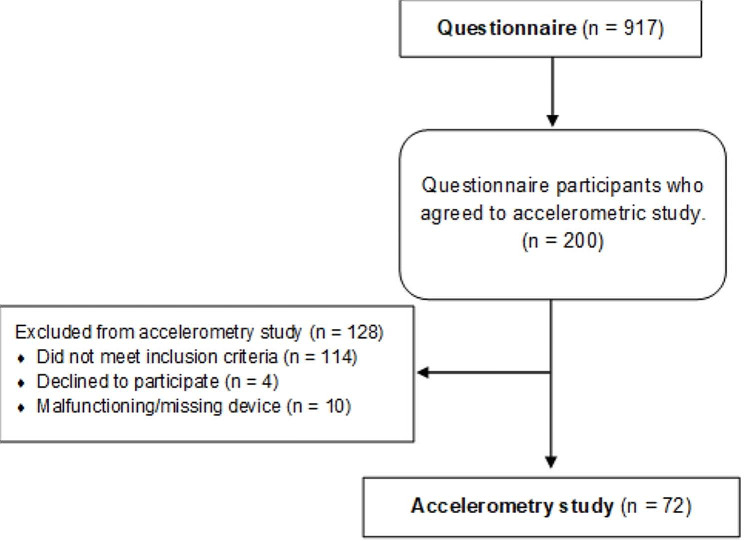
Flowchart of the study participants

To be included in the current study the participants needed to be receiving initial support, defined as food distribution and/or security alarms, from municipal caregivers. Residents with known cognitive impairments (e.g., diagnosed with dementia) and/or known wheelchair dependence were excluded from the study. Of the 917 questionnaire respondents, 200 agreed to participate in the accelerometry study. Of the 200 respondents, 114 were excluded due to not meeting the inclusion criteria mentioned above, and four chose to leave the study. Among the 82 participants who wore an accelerometer, ten accelerometers suffered technical errors, resulting in a study population of 72 older adults (Fig. 1). Compared with the large study sample, more of those who agreed to wear an accelerometer reported an intention to increase their physical activity level (*p* = 0.020) and a larger proportion was found to be in the high category of the Relative Autonomy Index (*p* = 0.039) (Supplementary file 1).The study was performed in line with the Declaration of Helsinki [27] and received approval from the Swedish Ethical Review Authority, Dnr 2020 − 00306. All participants gave verbal and written informed consent and received written information about the study.

### Assessment of physical activity

An information meeting was held with the participants who agreed to be involved. During the meeting, information was provided and verbal and written consent were collected. Mounting of accelerometers, ActivPAL 3 (Glasgow, Scotland), was done by a trained researcher. The accelerometer was encapsulated in a waterproof rubber tube, and then attached at mid-thigh with Tegaderm™ (3 M Svenska AB, Solna, Sweden). Instructions were given on the importance of writing notes in case of removal, describing time, duration, and reason for removal, such as discomfort or taking a bath. Reattachment supplies were given. The first and last of the 9 days that the accelerometers were worn were excluded from the analysis, to reduce wearable awareness [28]. After the last day, the accelerometers were sent to the research team in postage-paid envelopes for analysis of data.

Returned accelerometer data were processed through the ActivPAL™ software by exporting *Events file* using Pal Analysis software. All ActivPAL™ measures were triaxial and following totals of physical activities were obtained through the ActivPAL software: time spent lying, sitting, standing and stepping. From the totals daily steps, sitting bouts of at least 30 and 60 min were summarized in Microsoft® Excel® and then converted to IBM® SPSS®. Invalid data were removed, with previous studies used to set valid wear time for study inclusion to at least 4 days with 10 or more waking hours a day [29–31]. The standing time and walking time measures available in the ActivPAL 3 software showed overlap with time spent in activity, which could be explained by the user being upright while standing and walking. To reduce bias, standing time and walking time were excluded. Instead, sitting and upright time, based solely on posture, were used to map the waking hours of the older adults. This is the most common way to use ActivPAL 3 and aligned with the study aim, supporting this exclusion [32, 33].

After data cleaning, each ActivPAL™ variable (i.e., sitting bouts of at least 30 and 60 min and daily steps) was averaged across the number of valid days and used for analysis.

### Measures

#### Outcome

Physical activity was measured objectively using accelerometers. Daily steps was chosen as the outcome for the study, as it is a suitable measure of daily physical activity in the relevant age group [23, 32, 34–36].

#### Predictors

*Sitting in bouts of > 60 min* was obtained through predefined settings. Sex was categorized as Male or Female. Self-rated health (ranging from “bad,” “fair,” “good,” “very good” to “excellent”) was categorized as “Bad” (bad to fair) or “Good” (good to excellent). Walking independently outdoors (No/Yes) was based on the need of walking aids or not. Intending to become more physically active (No/Yes) was based on questionnaire data. Fear of falling and Exercise motivation regulation were index-based and were taken from the questionnaire (more details below).

*Fear of falling*: The short Falls Efficacy Scale International (short FES-I) was used to measure fear of falling in everyday situations. The older adults indicated how worried they were about falling in seven everyday situations, with four response alternatives from “Not concerned at all” to “Very concerned” [37, 38]. Delbaere, Close [38] have concluded that total scores [7–28] on the questionnaire can be divided into three categories: 7–8 points is a low fear of falling, 9–13 points is a moderate fear of falling and 14–28 points is a high fear of falling. The FES-I has been validated for usage on older adults with no to moderate cognitive impairment [39]. Fear of falling was categorized as “Low” (low to moderate fear of falling) or “High” (high fear of falling).

*Exercise motivation regulation*: The Swedish version of Behavioral Regulation in Exercise Questionnaire-2 (BREQ-2) was used to measure motivation to exercise and physical activity with motivation profile [40]. BREQ-2 includes 19 statements about physical activity with five response alternatives ranging from “Not very true” to “True.” The subcategories of BREQ-2 are Amotivation (minimum value = 0, maximum value = 16), External regulation (minimum value = 0, maximum value = 16), Introjected regulation (minimum value = 0, maximum value = 12), Identified regulation (minimum value = 0, maximum value = 16), and Intrinsic regulation (minimum value = 0, maximum value = 16). To calculate the older adults’ motivation regulation, the Relative Autonomy Index (RAI) was used. The RAI scale ranges from − 24 to + 20, where a higher positive score indicates greater relative autonomy. The index was calculated by weighting the subcategories [8, 41]. RAI score was categorized as “low regulation” (below average within the group) or “high regulation” (average or above average within the group).

### Statistical analysis

Descriptive statistics for personal characteristics and daily steps are presented as frequencies and percentages for categorical variables. T-tests were used to compare data at interval level: distribution of personal characteristics and daily steps between groups [42]. In the crude analysis, we examined the bivariate association between daily steps and the predictors using simple linear regression. We fitted a multiple linear regression with daily steps as the outcome, and sex, self-rated health, walking independently outdoors, intending to become more physically active, fear of falling, sitting in bouts of > 60 min per day (Sitting in bouts of > 30 min per day excluded due to bad model fit) and exercise motivation regulation as predictors. Due to missing data in the included predictors, the linear analysis included 66 subjects. After logarithmic transformation of the dependent variable, the model satisfied the assumption of normally distributed residuals and had no signs of heteroscedasticity or collinearity (all variance inflation factors were around 1). To investigate the independent contribution of each predictor, a series of block-wise linear regression models was fitted. The magnitude of the change to $$ {R}^{2} $$was interpreted as the unique contribution of each predictor to the model and gave an idea of the relative importance of the predictors [43]. The outcome of the regression model is presented with standardized beta (β), confidence interval, *p*-value (p), and R-squared ($$ {R}^{2}$$), as recommended for statistical interpretation [44]. All tests were two-tailed with a significance level set at *p* = 0.05. Data were processed using IBM® SPSS® version 27.0.

## Result

The participants included 44 females (median age 83 years, range 21) and 28 males (median age 83 years, range 24). Self-rated health was reported as less than good in 54% of the sample, non-independent outdoor movement were the case for 61%, and moderate to high fear of falling was reported by a majority (75%) of the group. The mean time of sitting was 10.15 h a day (SD 2.10) and the average step count was 4,329 steps (SD 2,337). Of the older adults 65% had a high motivation to exercise, and 53% had the intention to increase physical activity levels (Table 1).

**Table 1:** Sample characteristics and distribution of total number of daily steps

	N	%	Steps (Mean)	Standard deviation	*p* ^a, c^
Sex					0.323
Female	44	61	4,547	2,310	
Male	28	39	3,985	2,379	
Age					0.334
70–83 years	38	53	4,076	2,058	
84–97 years	34	47	4,611	2,616	
Self-rated health					0.009
Good	33	46	5,100	2,314	
Less than good	39	54	3,676	2,177	
Outdoor movement					0.006
Independent	28	39	5,267	2,252	
Non-independent	44	61	3,732	2,212	
Fear of falling					0.478
Low	18	25	4,671	2,415	
Moderate to high	54	75	4,215	2,322	
Intention to increase physical activity					0.002
Yes	35	53	3,518	2,034	
No	31	47	5,259	2,433	
Relative autonomy index (RAI)					0.026
High level of motivation regulation for exercise	47	65	4,772	2,420	
Low level of motivation regulation for exercise	25	35	3,495	1,953	
Sitting bouts of > 60 min per day					< 0.001
Two or less	47	65	5,035	2,227	
Three or more	25	35	3,001	1,959	
Total	72^b^	100 ^b^	4,329	2,337	

^a^*p*-value for the mean difference between groups calculated with independent T-test

^b^ Total numbers are not equal to 72 or 100% due to missing data in some variables

^c^ Confidence interval 95%

In the crude analysis (Table 2), having high self-rated health, walking independently outdoors, and being motivated for physical exercise were positively associated with daily steps. Among the subgroups regarding motivation for physical exercise, identified regulation and intrinsic regulation were positively associated with daily steps, while bouts of at least 60 min of sitting per day and intending to become more physically active were negatively associated with daily steps.

**Table 2:** Crude and adjusted analysis

	Crude analysis			Adjusted analysis			
	β^b^	Confidence interval 95%^b^	p^b^	β^c^	Confidence interval 95%^c^	p^c^	$$ {R}^{2}$$ ^c^
**Constant** ^**a**^						˂ 0.001	
Sex	0.147	-0.051 - 0.222	0.216	0.279	0.053 - 0.261	0.004	0.008
Self-rated health	0.298	0.040 - 0.298	0.011	0.213	0.007 - 0.232	0.038	0.076
Walking independently outdoors	0.324	0.057 - 0.319	0.005	0.301	0.063 - 0.281	0.003	0.092
Intending to become more physically active	-0.379	-0.340 - -0.083	0.002	-0.294	-0.279 - -0.049	0.006	0.130
Fear of falling	0.091	-0.096 - -0.214	0.447	-0.238	-0.282 - -0.022	0.023	-0.006
≥ 60 min sitting bouts	-0.455	-0.396 - -0.144	˂ 0.001	-0.313	-0.300 - -0.690	0.002	0.196
Relative autonomy index	0.407	0.002 - 0.006	˂ 0.001	0.258	0.044 - 0.268	0.007	0.076
Amotivation	-0.086	-0.030 - 0.014	0.474				
External regulation	-0.142	-0.042 - 0.010	0.232				
Introjected regulation	0.095	-0.012 - 0.027	0.426				
Identified regulation	0.333	0.006 - 0.029	0.004				
Intrinsic regulation	0.409	0.009 - 0.031	˂ 0.001				
**Total explained variance (R²)**							**0.478**

^a^ Total number of daily steps converted into a continuous variable, ^b^ Bivariate regression, ^c^ Multiple linear regression

Reference values: sex (male = 0, female = 1), self-rated health (bad or fair = 0, good = 1), walking independently outdoors (no = 0, yes = 1), intending to become more physically active (no = 0, yes = 1), fear of falling (low = 0, moderate to high = 1), ≥ 60 min bouts of sitting (two times or less per day = 0, three times or more per day = 1), relative autonomy index (low level of motivation regulation for exercise = 0, high level of motivation regulation for exercise = 1)

In adjusted analyses (Table 2), the model explained 47.8% of the variance in daily steps. Out of the seven predictors, time spent sitting in bouts of at least 60 min per day ($$ {R}^{2}$$ = 19.6%), independence when walking outdoors ($$ {R}^{2}$$ = 9.2%), and intention to become more physically active ($$ {R}^{2}$$ = 13.0%) explained daily steps to the greatest degree.

## Discussion

Using device-measured daily steps together with subjective survey measures of physical function and daily physical activity among older adults living at home with initial support, we found predictors explaining 47.8% of the variation in daily steps among community-dwelling older adults receiving initial support. In this study, sitting in fewer bouts of at least 60 min per day, walking independently outdoors, and intending to become more physically active were the most important predictors of daily steps.

The correlation between physical activity and self-rated health is well-known [45]. Lower levels of physical activity is a predictor of long-term mortality [46], cognitive impairment [47], and frailty for older adults [48]. Intuitively, it is attractive to interpret our findings and previous findings as suggesting that an increase in physical activity will reduce the effects of poor health. However, reverse causality cannot be ruled out [49], since frailty connected to advanced age is inevitable and may greatly decrease physical capacity, autonomy in daily living, and the endurance needed for maintaining a desired level of physical activity WHO [1]. Our findings regarding health and physical activity, in terms of increased daily steps, are consistent with those of Paluch, Bajpai [25]. Regarding motivation, Brunet and Sabiston [50] highlighted the importance of examining identified and intrinsic regulation for physical activity in order to understand why we engage in activity and intend to be more active. This inherent drive is a source worth looking into more closely. According to Booth [51], the identified regulation can play a significant role when it comes to older adults’ motivation. Our findings indicate that older adults are strongly motivated to engage in physical exercise but at the same time 35% indicates the lack of motivation needed to maintain physical exercise– understanding identified and intrinsic regulation could therefore be important.

A recommendation to break prolonged sitting every thirty minutes [52] has been given for a decade, but recent findings highlight the need for more research regarding recommended duration of time spent sitting [53]. Both thirty-minute bouts and sixty-minute bouts were included in the initial analysis. However, the thirty-minute bouts was excluded from further analysis due to bad model fit. Our study indicates that for older adults living at home with initial support, a suggestion could be to not sit for more than sixty minutes more than two times per day, if the intention is to increase daily steps. The importance of reducing the number of bouts of sitting for 60 min or more per day in older adults is a finding related to previous research [53].

The older adults in the present study who intended to become more physically active were the ones who were below average daily steps, indicating that older adults might have great insight into their daily physical activity. This could be especially relevant as the older adults who did not intend to become more physically active had a mean step count at 5,259, whilst the total mean step count was 4,329 (Table 1). Though the daily steps may seem low, this is a finding that underlines the importance of addressing physical activity intensity, rather than setting a specific number as an absolute goal [54, 55]. Older adults who had a motivation regulation below identified regulation might have been at higher risk of becoming sedentary due to a lack of consistency in their daily physical activity. Reducing sedentary behavior is harder to do without support, especially for older adults who have a low degree of self-determination [8]. If we add the fact that the bivariate analysis of RAI showed a positive association with the number of daily steps, interest should increase for deeper analysis of the regulation of motivation for physical exercise in the older population. Indexing motivation to study the regulation of exercise motivation has been a focus of discussions, as the behavioral patterns might be too complex to analyze with a single scale [56]. Therefore, in the present study, the analysis of regulation was based on the subcategories in the scoring of motivation, acting as a linear predictor of the correlation with daily steps. Motivation, with its complex nature, requires further research, in two areas in particular. The inherent drive for physical activity needs to be more thoroughly analyzed to allow comprehension of how older adults get this drive. The second area is why amotivation for exercise does not seem to impact daily steps.

### Strengths and limitations

The findings of Hyde, Nguyen [36] address daily steps as a blunt measure in that the threshold for a step is not always reached even if a step is taken and that all commonly used accelerometers may therefore underestimate daily steps in older adults. Though daily step count is a relatively blunt measure of physical activity, as it does not capture intensity, it can still be of great value since it is equally blunt for all participants. Moreover, if one assumes that all participants had the same systematic reduction in daily steps due to lower sensitivity in the devices, only the absolute values would change– not the strength or direction of the regression coefficients [42, 57].

To provide the best fit of the multiple regression analysis, daily steps were computed to a continuous variable [42]. Age revealed no significance in the T-test, so its exclusion was anticipated. However, the indication that age does not have an impact on daily steps is interesting as aging has robust associations with mobility limitations [1, 58–60]. One assumption could be that it is not age itself, but factors related to aging, that affect physical activity among older adults [5–7].

Our sample was relatively small, but the drop-out analysis shows that it was similar to the population of the larger study. However, the difference in intention to become more physically active and the RAI score indicates that our sample was likely to be slightly more active than the larger population, though differences in associations may remain the same.

## Conclusion

The model of predictors promotes a new understanding to daily steps among community-dwelling older adults. The association between sitting in bouts of 60 min and daily steps and the impact of independence when walking outdoors can prove useful information for clinical practitioners devising interventions to break prolonged sitting. Future research should prioritize studying the outdoor walking independence of older adults, defining it in relation to walking with or without assistive devices, and understanding its impact on physical activity and sedentary behavior.

### Electronic supplementary material

Below is the link to the electronic supplementary material.

**Supplementary file 1**. Comparison of the large study sample and those who agreed to wear an accelerometer.

## Data Availability

The datasets generated and/or analyzed during the current study are not publicly available due to governing laws in Europe and the agreement between participants and the research group regarding data management, but are available from the corresponding author on reasonable request.

## Abbreviations

β: Standardized beta
p: *p*-value
R^2^: Coefficient of determination for variance in linear regression model
FES-I: Falls Efficacy Scale International
RAI: Relative autonomy index
BREQ-2: Behavioral Regulation in Exercise Questionnaire-2
