# Supplementary material for: Daily activity patterns in older adults receiving initial support: the association between daily steps and sitting in bouts of at least 60 min
Source: BMC Geriatr. 2024 Jan 23;24:88. doi: 10.1186/s12877-024-04681-3 (PMC10807219; doi:10.1186/s12877-024-04681-3)
Supplement: Supplementary file 1 — Comparison of the large study sample and those who agreed to wear an accelerometer. [file 12877_2024_4681_MOESM1_ESM.docx]

|  | Those who agreed to wear an accelerometer  (N = 72) | Large study sample  (N = 917) | p ^a^ |
| --- | --- | --- | --- |
| Sex  Male  Female |  |  | 0.899 |
|  | 28 (39%) | 261 (33.%) |  |
|  | 44 (61%) | 527 (67%) |  |
| Age  70–83 years  84–97 years |  |  | 0.082 |
|  | 38 (53%) | 307 (41%) |  |
|  | 34 (47%) | 440 (59%) |  |
| Self-rated health  Bad  Good |  |  | 0.102 |
|  | 39 (54%) | 543 (67%) |  |
|  | 33 (42%) | 269 (33%) |  |
| Outdoor movement  Non-independent  Independent |  |  | 0.193 |
|  | 44 (61%) | 436 (54%) |  |
|  | 28 (39%) | 371 (46%) |  |
| Fear of falling  Moderate to high  Low |  |  | 0.444 |
|  | 54 (75%) | 692 (83%) |  |
|  | 18 (25%) | 144 (17%) |  |
| Intention to increase physical activity  No  Yes |  |  | 0.020 |
|  | 31 (43%) | 425 (58%) |  |
|  | 35 (57%) | 305 (42%) |  |
| Relative autonomy index (RAI)  Low level of motivation regulation for exercise  High level of motivation regulation for exercise |  |  | 0.039 |
|  | 25 (35%) | 225 (49%) |  |
|  | 47 (65%) | 238 (51%) |  |
| Total | 72 (100%) ^b^ | 917 (100%) ^c^ |  |

**Supplementary file 1.** Comparison of the large study sample and those who agreed to wear an accelerometer.

^a^ p-value for the mean difference between groups calculated with independent T-test. ^b^ Total numbers are not equal to 72 or 100% due to missing data in some variables.
^c^ Total numbers are not equal to 917 or 100% due to missing data in some variables.
